# Parental palliative cancer: psychosocial adjustment and health-related quality of life in adolescents participating in a German family counselling service

**DOI:** 10.1186/1472-684X-11-21

**Published:** 2012-10-30

**Authors:** Franziska Kühne, Thomas Krattenmacher, Corinna Bergelt, Johanna C Ernst, Hans-Henning Flechtner, Daniel Führer, Wolfgang Herzog, Kai v Klitzing, Georg Romer, Birgit Möller

**Affiliations:** 1 Department of Child and Adolescent Psychiatry, Psychotherapy, and Psychosomatics, University Medical Centre Hamburg-Eppendorf, Martinistraße 52, Hamburg, 20246, Germany; 2 Department of Medical Psychology, University Medical Centre Hamburg-Eppendorf, Martinistraße 52, Hamburg, 20246, Germany; 3 Department Of Child and Adolescent Psychiatry, Psychotherapy and Psychosomatics, Otto-von-Guericke University, Leipziger Str. 44, Magdeburg, 39120, Germany; 4 Department of Child and Adolescent Psychiatry, Psychosomatics, and Psychotherapy, Charité University Medical Centre, Augustenburger Platz 1, Berlin, 13353, Germany; 5 Department of Psychosomatic and General Clinical Medicine, University Medical Centre, Im Neuenheimer Feld 325, Heidelberg, 69120, Germany; 6 Department of Child and Adolescent Psychiatry, Psychotherapy, and Psychosomatics, University Medical Centre, Liebigstraße 20a, Leipzig, 04103, Germany

**Keywords:** Palliative care, Adolescents, Psychosocial adjustment, Health-related quality of life, Coping

## Abstract

**Background:**

Parental palliative disease is a family affair, however adolescent's well-being and coping are still rarely considered. The objectives of this paper were a) to identify differences in psychosocial adjustment and health-related quality of life (HRQoL) among adolescents and young adults with parents suffering from palliative cancer or cancers in other disease stages, b) to relate psychosocial adjustment and health-related quality of life to adolescent coping, and c) to explore significant mediator and predictor variables.

**Methods:**

Cross-sectional data were derived from a multi-site research study of families before child-centered counselling. N=86 adolescents and young adults were included, their mean age 13.78 years (sd 2.45), 56% being female. Performed analyses included ANCOVA, multiple linear regression, and mediation analysis.

**Results:**

Adolescents with parents suffering from palliative cancers reported significantly less total psychosocial problems, and better overall HRQoL. There were no significant group differences regarding coping frequency and efficacy. Our set of coping items significantly mediated the effect of parental disease stage on psychosocial problems and HRQoL. Further, parental disease status and general family functioning predicted psychosocial problems (R^2^_adj_ =.390) and HRQoL (R^2^_adj_ =.239) best.

**Conclusion:**

The study indicates distress among adolescents throughout the entire parental disease process. Our analysis suggests that counselling services could offer supportive interventions which focus particularly on adolescent coping as well as family functioning.

## Background

"The family is the centerpiece of palliative care in cancer"(p. 439) [1], and parental cancer affects the psychological well-being of patients, spouses, and children [1-3]. Regarding the percentage of minor children of cancer patients, estimations with differing reliability vary between 5-15% [4], 24% (see [5]), and 30% [6]. According to a US population-based estimate, 18% of cancer patients diagnosed within the last 2 years live with minor children [7]. But when families are confronted with a life-threatening parental disease, dependent children are still a "forgotten group" (p.459) [8].

Considering recent reviews in the field, the following variables seem to be the ones which are theoretically best supported, regarding children's adjustment to parental illness, especially cancer: children's age [4,9-12], and gender [4,9,11-13], gender of the ill parent [9,12,13], and psychological functioning (esp. depressive symptoms) of the ill parent [10,12]. Family functioning appears to be a further important factor [12,14-16]. Two reviews in the field of parental cancer report type of illness [4,10], illness duration [10], and disease severity [4] to be of importance, but still most studies concentrate on cancer in general [4,10,12], or early disease stages [11].

As expected in parental palliative situations, adolescents stated that they were more stressed than adolescents whose parents survived cancer [17]. They expressed their fear, sadness, and anxiety regarding parental palliative disease and death [18], and their adjustment seems to be influenced by disease and treatment burden [19]. Although parental palliative disease is regarded as a period of high risk for children's psychological vulnerability [20], there is a knowledge gap concerning quantitative studies on dependent children of palliative cancer patients [21].

We therefore conducted a questionnaire study investigating the psychosocial impact of parental cancer in palliative vs. other disease stages on adolescents and young adults. Since several studies indicate a relation between coping and HRQoL [22-24], but as there is little knowledge regarding coping of children of palliative patients [25], we explored adolescents’ coping in relation to our main outcomes. Based on the theoretical model proposed by Su & Ryan-Wenger [9], we tested a mediation hypothesis linking parental disease stage to adolescent's psychosocial adjustment through adolescent's coping. Further, we analysed group differences between adolescents and young adults of palliative patients vs. patients in other disease stages, and considering the empirical studies mentioned above, we hypothesised adolescents with parents suffering from palliative cancer to be characterised by more psychosocial problems and lower HRQoL. Beyond, we explored variables as potential predictors of our outcome criteria (psychosocial problems and HRQoL).

## Methods

### Design and sample

Our study is part of a German multisite research project, including data of five study centres in Berlin, Hamburg, Heidelberg, Magdeburg, and Leipzig. The sample consisted of families with parents suffering from cancer participating in child- and family-centred counselling for "Children of Somatically Ill Parents" (COSIP [26]). All centres provided low-threshold counselling due to the common COSIP concept, and counsellors received regular joint training.

In our cross-sectional study, we focused on the first measurement point (t_1_) of the longitudinal multisite data set. After registration for counselling, families were called and asked for study participation (informed consent). Data was collected from September 2009 - June 2011, and questionnaires were filled out by family members at home before counselling. In all centres, ethical approvals of the respective institutions were obtained (Ethik-Kommission der Ärztekammer Hamburg, reference number PV3322; Ethik-Kommission der Otto-von-Guericke-Universität an der Medizinischen Fakultät und am Universitätsklinikum Magdeburg, reference number 56/09; Ethikkommission der Charité - Universitätsmedizin Berlin, Ethikausschuss 2 am Campus Virchow-Klinikum, reference number EA2/096/08; Ethikkommission Medizinische Fakultät Heidelberg, reference number S-030/2009; Ethikkommission an der Medizinischen Fakultät der Universität Leipzig, reference number 358–2008).

Inclusion criteria were parental cancer, and at least one child ≤ 21 years old. Families with a parent suffering from psychotic disease, acute self or extrinsical threat, or insufficient German language skills were excluded. If the patient was severely ill and could not participate, only questionnaires of the healthy parent and/or children were considered. We defined palliative patients (based on [27]) as patients with limited life expectancy, whose disease was considered non-curable by parents themselves, or by medical personnel (physician or psycho-oncologist) at the first counselling encounter. This definition extends a mainly physician-based assessment in order to adapt to our counselling context. Families of patients whose disease status was unknown or those who died before the first counselling contact were excluded from our analyses.

### Instruments

#### SDQ

The Strength and Difficulties Questionnaire [28] (SDQ) is a questionnaire screening behavioural strengths and problems of 4–16 year olds. 25 items form five dimensions (emotional problems, behavioural problems, hyperactivity, peer relationship problems, pro-social behaviour). For the German self-rating version, Cronbach's alpha was .78 in an in- and outpatient psychiatric sample, and discriminative validity was comparable to that of another established measure [29].

#### Kidscreen

The KIDSCREEN-10 [30] is an unidimensional questionnaire measuring global well-being and health-related quality of life of 8–18 year old children and adolescents providing a singular global health index. It has good psychometric properties, with Cronbachs alpha r=.82 (internal consistency), ICC=.70 (2-weeks retest-reliability), and moderate to high correlations with other HRQoL questionnaires (convergent validity) [31]. Additionally, we involved the five Kidscreen-27 HRQoL subscales (physical well-being, psychological well-being, autonomy & parent relation, social support & peers, school environment), with reported internal consistencies ranging from 0.80 to 0.84.

#### Kidcope

The Kidcope [32,33] is a checklist assessing frequency, and respective efficacy of the use of cognitive and behavioural coping via 11 items (Table 1). It can be administered to children of 7–19 years old. There is moderate internal consistency of the German version (r = .30-.70). Due to low Cronbach's alphas of potential subscales in our sample (r < .30), we did not distinguish active from passive strategies, but analysed on a single item basis.

**Table 1:** Demographic characteristics of patients and adolescents

**Patient characteristics (N=49)**		**Adolescent characteristics(N=86)**		
Mean age 44.31 (sd 6.40; range 29–65)		Mean age 13.78 (sd 2.45; range 11–21)		
Female	57.1% (n=28)	Female	55.8% (n=48)	
Cancer (n=49)		Age groups (n=86)		
various cancers^1^	55.1% (n=27)	Early adolescence (11–14 years)		64.0% (n=55)
breast / gynecol.	26.5% (n=13)	Late adolescence (15–18 years)		31.4% (n=27)
digestive organs	18.4% (n=9)	Young adulthood (19–21 years)		4.6% (n=4 )
First diagnosis (n=48)				
0-12 months ago	45.8% (n=22)	Study centre^2^	Parents in palliative situation (n=43)	Parents in other disease stages (n=43)
>12 months ago	54.2% (n=26)			
Specialised palliative care (n=47)				
yes	12.8% (n=6)	Heidelberg-PM	n=14 (16.3%)	n=13 (15.1%)
no	87.2% (n=41)	Berlin-CAP	n=9 (15.1%)	n=13 (10.5%)
ECOG performance status^4^ (n=49)		Hamburg-CAP	n=13 (10.5%)	n=9 (15.1%)
0-1	61.2% (n=30)	Leipzig-CAP	n=7 (8.1%)	n=6 (7.0%)
2-4	38.8% (n=19)	Magdeburg-CAP	-	n=2 (2.3%)

Note: ^1^ like blood or lung cancers (cell sizes each n<4); ^2^ PM (Psychosomatic Medicine), CAP (Child and Adolescent Psychiatry); ^3^ WHO-ECOG performance status from 0 (asymptomatic) to 4 (completely disabled).

#### HADS

The Hospital Anxiety and Depression Scale [34] assesses symptoms of anxiety and depression in physically ill patients on 4-point-scales. The authors indicate Cronbach's alpha with .80 (anxiety) / .81 (depression) in their sample of predominantly cardiological patients.

#### FAD

Family functioning was measured by Family Assessment Device [35] (FAD), which is a self-report measure collecting information on the family system as a whole. Out of the FAD subscales, we used the general functioning (gf) subscale, which assesses overall health/pathology of family functioning, for our analyses. Higher scores (> 2) may indicate family dysfunction [36]. Internal consistency of the gf-scale was .92 in a psychiatric [35] and .89 in an oncological sample [37].

### Statistical analyses

Analyses were performed using SPSS 16.0. We calculated descriptive statistics, Chi^2^-tests for categorial data, t-tests for norm comparisons, Pearson correlation coefficients, ANCOVAs (covariate COV = child's age), and multiple linear regressions (backward). If applicable, predictors were dummy coded, and significance levels adjusted by Bonferroni correction.

For mediation analysis, we referred to Preacher & Hayes' [38] multiple mediator model applying their SPSS macro [39]. Kidcope frequency items showing significant Pearson correlation coefficients with psychosocial adjustment (Items 2, 4, 7, 9, 10; see Table 1) were included as mediators. Because of item intercorrelations, only the total indirect effect was interpreted, bias-corrected (BC) Bootstrapping (1000 bootstrap samples) was used for obtaining confidence limits, and bias-corrected and accelerated (BCa) CIs were reported.

Since in 73% only one child per family answered the questionnaires, there was not enough variance to account for the family factor within a mixed linear model as our SPSS analysis showed.

In the original data set, there were significantly more missings in the SDQ (19.5%) and Kidscreen ratings (22.0%) of children of patients with other disease stages than with palliative stage cancers (each *p *= .001). There were no significant missing differences concerning child age and gender, Kidcope frequency (20.3%-22.8%) and subordinate efficacy (22.0%-29.3%).

State-of-the-art imputation strategies (EM algorithm, multiple imputation) require larger than usual sample sizes [40,41]. On the other hand, single imputation may also lead to biased data sets and estimates. Therefore, in order to obtain a stable data set, we chose a conservative method in referring to a set of complete data in SDQ, Kidscreen, and Kidcope frequency. We compared our findings to those of the original data set, whereas there were no differing results.

## Results

### Psychosocial problems

For demographic data of n=86 adolescents nested within N=66 families see Table 1. As there are no German normative data [42], we compared the bandings of the total difficulties scores with those of a British community sample of 83 11–16 year olds [43]. In our overall sample, 74.4% (n=64) vs. 77% of the adolescents in the community sample reported scores in the normal range, 10.5% (n=9) vs. 18% reported borderline, and 15.1% (n=13) vs. 5% abnormally high scores.

There was no significant difference comparing SDQ total problems between children of palliative patients (mean 10.23, sd 5.21) with the British comparison sample (T=−1.470(df=42), *p * = .149). Children of patients in other disease stages (mean 14.95, sd 5.35) reported significantly more psychosocial problems (T=3.460(df=42), *p *< .001) compared to the community sample. The result persisted if we limited our sample to 11–16 year olds in accordance with the age range of the comparison sample.

Children of palliative patients reported significantly less total (F=16.246(df=1, 83); *p *< .001, 95% CI [−6.892, -2.338], partial eta^2^=.164, child's age *p *= .360), emotional (F=24.961(df=1, 83); *p *< .001, 95% CI [−3.299, -1.420], partial eta^2^=.231, child's age *p *= .825), and behavioural problems (F=9.518(df=1, 83); *p *= .003, 95% CI [−1.666, -.360], partial eta^2^=.103, child's age *p *= .087) than children whose parents were in other disease stages.

### Health-related quality of life (HRQoL)

In a German normative sample of 1.723 8–18 year olds, 97.4% described their health as good, very good or excellent [44]. In our overall sample, this was true for 79.8% (n=67) adolescents. 20.2% (n=17) characterised their health as fair or poor.

There was no significant difference between children of palliative patients (mean 48.70, sd 9.52) and 1.072 12–18 year old adolescents of a German normative sample (T=−1.035(df=42); *p *= .307). Adolescents with parents in other disease stages (mean 43.44, sd 7.13) reported significantly worse HRQoL than the norm sample (T=−6.212(df=42); *p *< .001). Again, the results persisted if we only included 12–18 year old adolescents.

Children of palliative patients reported significantly better overall HRQoL (F=10.776(df=1, 83); *p *= .002, 95% CI [2.266, 9.235], partial eta^2^=.115), physical well-being (F=8.380(df=1, 83); *p *= .005, 95% CI [1.766, 9.542], partial eta^2^=.092), psychological well-being (F=10.230(df=1, 83); *p *= .002, 95% CI [2.474, 10.609], partial eta^2^=.110), and school environment (F=7.783(df=1, 81); *p *= .007, 95% CI [1.475, 8.809], partial eta^2^=.088) as compared with children whose parents were in other disease stages. Regarding total (B = −1.018; *p *= 0.008; partial eta^2^=.088) and physical well-being (B = −1.284; *p *= 0.002; partial eta^2^=.110), older age groups reported significantly lower HRQoL.

There were no significant differences in psychosocial problems regarding adolescent's age and gender, and no significant differences in HRQoL regarding adolescent's gender (*p *> .025).

### Behavioural and cognitive coping

**Figure 1:**
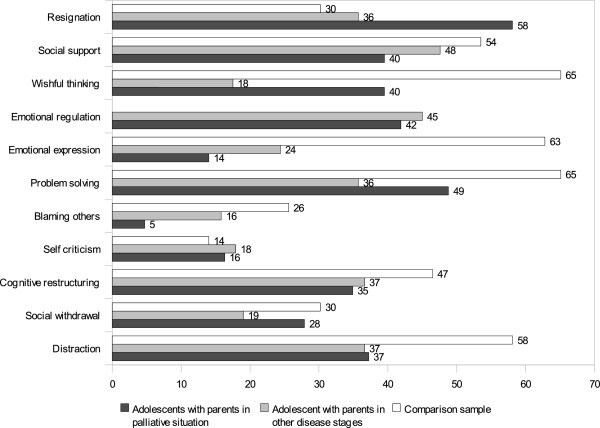
**Kidcope frequency of the categories mostly** / **almost all the time in%.** Note: n=43 answering each item, comparison sample [33].

**Figure 2:**
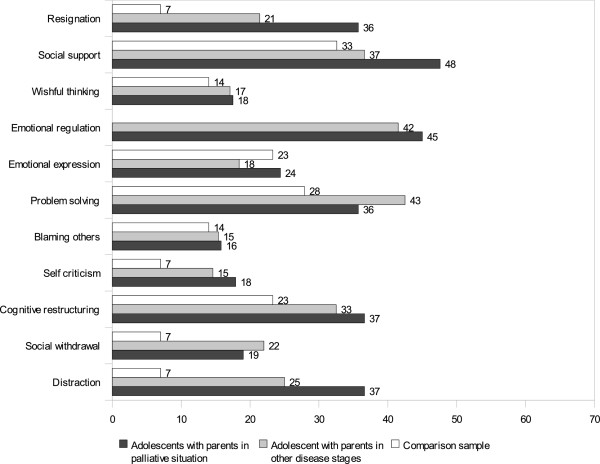
**Kidcope efficacy of the categories mostly/almost all the time in%.** Note: n=38-43 answering each item, comparison sample [33].

Although the frequencies suggest differences between adolescents of the two groups (Figures 1, 2), none was statistically significant (*p*>.0045). Regarding child age and gender, there were no significant group differences as well. In comparison with 43 German 7–19 year old diabetes patients facing a common problem [33], adolescents in our sample used most strategies less often, but perceived most strategies to be more efficacious. Adolescents of palliative patients perceived distraction and resignation as efficacious strategies. Bivariate correlations between coping and psychosocial adjustment are shown in Table 2.

**Table 2:** Pearson correlations of psychosocial adjustment and coping

**Kidcope item**		**Abbreviation**	**Frequency(n=86)**		**Efficacy(n=79-84)**	
			**Kidscreen**	**SDQ**	**Kidscreen**	**SDQ**
1	I just tried to forget it / I did something like watch TV or played a game to forget it	Distraction	-.137	.170	.212	-.183
2	I stayed by myself / I kept quiet about the problem	Social withdrawal	-.286**	.225*	.107	-.138
3	I tried to see the good side of things	Cognitive restructuring	.183	-.126	.216	-.188
4	I blamed myself for causing the problem	Self criticism	-.294**	.417**	.146	.011
5	I blamed someone else for causing the problem	Blaming others	.001	.119	.181	.016
6	I tried to fix the problem by thinking of answers / doing something or talking to someone	Problem solving	.110	-.191	.250*	-.308**
7	German: I spoke about how I felt / I yelled, screamed, or got mad	Emotional expression	-.258*	.354**	-.058	.200
8	I tried to calm myself down	Emotional regulation	-.055	.017	.136	-.076
9	I wished the problem had never happened / I wished I could make things different	Wishful thinking	.005	.263*	.255*	-.028
10	I tried to feel better by spending time with others like family, grownups, or friends	Social support	.294**	-.191	.357**	-.188
11	I didn't do anything because the problem couldn't be fixed	Resignation	.074	-.202	.194	-.252*
		Kidscreen and SDQ	-.584**			

Note: ** *p* < .01; * *p* < .05.

Taken as a set, Kidcope items 2 (Social withdrawal), 4 (Self criticism), 7 (Emotional expression), 9 (Wishful thinking), and 10 (Social support) significantly mediated the effect of parental disease stage on SDQ psychosocial problems (total indirect effect: point estimate 1.6352; SE=.6876; Z=2.3782; p = .0174; BCa 95% CI [.3077;2.9362]). The total and direct effects were 4.7209, p = .0001 and 3.0857, p = .0070 respectively. Similarly, our set of items mediated the effect of parental disease stage on Kidscreen HRQoL (total indirect effect: point estimate −2.3243; SE=1.0314; Z=−2.2536; p = .0242; BCa 95% CI [−4.5242;-.2741]), whereas the total and direct effects were −5,2537, p = .0048 and −2.9295, p = .1145.

### Multiple linear regression models

Predictors included in our regression analyses were parental disease status, adolescent age and gender, adolescent-rated FAD general functioning, ill parent's gender, and HADS depression of the ill parent.

Parental palliative disease was associated with lower psychosocial problems, and better HRQoL of adolescents (Table 3). Worse general family functioning predicted more psychosocial problems, and worse HRQoL. Further, older age was associated with lower HRQoL in our sample. The two models accounted for 39% (SDQ) resp. 24% (Kidscreen) of the variance.

**Table 3:** **Multiple linear regressions** (**n**=**63**)

**Criteria**	**F (df) **,***p***	**R**^**2**^	**R**^**2**^_**adj**_	**Significant predictors**	**B**	**95%CI for B**	**beta**	***p***	**VIF**
SDQ total difficulties	8.934 (5, 57), <.001	.439	.390	Parental palliative disease	−5.260	[−7.715; -2.805]	-.446	<.001	1.097
				FAD general functioning	4.639	[2.157; 7.121]	.383	<.001	1.064
				Adolescent age	−1.754	[−4.226; .718]	-.144	.161	1.038
				Adolescent gender	1.700	[−.732; 4.131]	.144	.167	1.076
				HADS-D ill parent	3.813	[−.622; 8.248]	.175	.091	1.053
Kidscreen-10 global health index	7.429 (4, 58), <.001	.339	.239	Parental palliative disease	4.805	[.637; 8.973]	.258	.025	1.092
				FAD general functioning	−6.396	[−10.695; -2.097]	-.334	.004	1.103
				Adolescent age	−4.751	[−9.068; -.438]	-.246	.031	1.091
				Gender ill parent	2.755	[−2.429; 7.939]	.123	.292	1.179

Note: Dummy coding of parental palliative disease (no vs. yes), FAD gf (< 2 vs. > =2), child age (11–14 vs. 15–18 years), VIF (variance inflation factor).

## Discussion

The current study focused on psychosocial problems and health-related quality of life of adolescents and young adults with parents suffering from palliative cancer versus cancer in other disease stages, and on associations of both outcomes with coping. As compared to other samples, more adolescents in our overall sample (15%) indicated abnormally high SDQ scores, and 20% characterised their health as fair or poor. But in contradiction to our hypothesis, adolescents whose parents were in other disease stages described themselves as having more psychosocial problems and worse HRQoL than adolescents with parents suffering from palliative cancer. Correspondingly, these adolescents reported more total, emotional, and behavioural problems, worse overall, physical, and psychological well-being, as well as more problems in their school environment than adolescents with parents in palliative situations. SDQ and Kidscreen scores of adolescents with parents suffering from palliative cancer were similar to those of the comparison samples.

Similarly in another study, forewarning of death was associated with poorer parent- and teacher-rated outcomes, but better child-rated outcomes [45]. As one explanation for these findings, cancer might be characterised by insecurity from the first diagnosis. From a care perspective, the findings of our study point to the need for psychosocial support for adolescents whose parents are suffering from cancer throughout the entire parental disease course.

Above, empirical studies show that children of cancer patients often conceal their thoughts in order to protect their parents [13], and it might be speculated if this might apply even more in parental palliative disease. Low self-report scores might further reflect the child's efforts to ward off potentially overwhelming emotions [45]. Child symptoms might occur later, past the most distressing period.

Beyond, parental disease status may not affect child psychosocial adjustment in isolation, but in association with other family aspects, like communication [46]. Parents often seek our counselling worrying how to talk with their children about disease deterioration. As our data illustrate the status at the beginning of counselling, our clinical experience shows that often, children were not informed about the palliative situation yet. Since patients still seem to be physically quite stable, changes might not have been evident in everyday life very much, and being (not) informed about parental disease status influences children's coping [13].

As opposed to other empirical results, we found no age and gender differences in psychosocial problems and coping, and no gender differences regarding HRQoL in our sample. The only age effect was related to total and physical well-being: older adolescent's reported decreasing HRQoL, which is a consistently observed empirical result [31,47].

Parental disease status was the most important predictor of psychosocial problems and HRQoL, and results interpretable in line with previous results. Again, older age was associated with lower HRQoL. Additionally, worse general family functioning predicted more psychosocial problems, and worse HRQoL. This is in accordance with the importance of family-related aspects documented in the literature [14,15].

The Kidcope is a commonly used short instrument assessing coping in a convenient way, but data are less detailed which influences psychometric properties [48,49]. As our mediator analysis showed, coping might mediate the influence of parental disease stage on adolescent's psychosocial adjustment, which should be analysed using further coping instruments in palliative care research.

Compared with diabetes patients, adolescents in our overall sample used most strategies less often, but perceived most strategies to be more efficacious which might be understood as a psychologically protective mechanism. Although there were no significant differences between adolescents of the two groups, children of palliative patients applied resignation more often and perceived this, just as distraction, as helpful strategies. Accordingly, in uncontrollable situations, disengagement / emotion-focused coping tends to be associated with better psychosocial adjustment [46]. Essential issues that should be considered in further studies in the field are perceived controllability and subjective appraisal of illness severity, and their influence on coping [50].

### Limitations

Our study comprised a convenience sample of middle and upper social class families seeking counselling. As adolescent's mean age was 14 years, it appears that our counselling did not reach older adolescents to the same extent. Physical and psychological well-being and family functioning are likely to vary in the course of the palliative phase which could be further elaborated by longitudinal designs. As we referred to cross-sectional data, the results of the mediational analyses do not allow for causal interpretations. Again, future work could build on our findings and examine these associations over time. In addition, studies on adolescents of palliative patients could include more sensitive or disease-specific items, maybe by combining the qualitative and quantitative approach.

## Conclusions

Adolescents with parents suffering from cancer are confronted with a critical life event which may raise psychological distress throughout the entire disease process. Primarily in parental palliative disease, adolescent resignation and distraction seem to be two of a diverse number of coping strategies used. Psychosocial counselling services could offer supportive interventions especially focusing on adolescent's individual resources, as well as enhancing meaning-focused coping strategies.

## Competing interests

The authors declare that they have no competing interests.

## Authors’ contributions

FK conceived the study, contributed to data acquisition, analysed and interpreted the data and drafted the manuscript. BM, CB, GR, JCE and TK made contributions to study conception, data acquisition and interpretation, and critically revised the manuscript. DF, HF, KVK and WH contributed to data acquisition, and critically revised the manuscript. All authors read and approved the final manuscript.

## Authors’ information

Georg Romer and Birgit Möller are the authors that shared last authorship.

## Pre-publication history

The pre-publication history for this paper can be accessed here:

http://www.biomedcentral.com/1472-684X/11/21/prepub
